# Ergonomic Risk Factors for Low Back Pain among Three-Wheel Drivers in Ethiopia: A Community-Based Cross-Sectional Study

**DOI:** 10.1155/2022/8133872

**Published:** 2022-02-04

**Authors:** Yonas Biratu Terfa, Adugna Olani Akuma, Ebissa Bayana Kebede, Abdisa Eba Tucho, Birhanu Abdisa, Selam Ayele, Mestawet Getachew Enbakom, Fikadu Balcha Hailu, Gugsa Nemera Germossa

**Affiliations:** ^1^School of Nursing, Institute of Health, Jimma University, Jimma, Ethiopia; ^2^Department of Nursing, Wollega University, Institute of Health Sciences, Nekemte, Oromiya, Ethiopia; ^3^School of Medicine, Institute of Health, Jimma University, Jimma, Ethiopia; ^4^School Pharmacy, Institute of Health, Jimma University, Jimma, Ethiopia

## Abstract

**Background:**

Driving a three-wheel car is an emerging job opportunity in most parts of developing countries. Drivers are at risk for developing low back pain (LBP). However, very little is known about the association between ergonomics factors and LBP among three-wheel drivers.

**Objective:**

This study was aimed to identify ergonomic risk factors of LBP among three-wheel drivers.

**Methods:**

*A community-based cross-sectional study on 396 participants was conducted in the Jimma city on all selected public three-wheel drivers in March, 2020. The data were collected using a standard questionnaire adapted from the Nordic Musculoskeletal questionnaire, anthropometric measurements, and observation checklist. The collected data were entered into Epi Data and exported to SPSS version 21.0. Logistic regression analysis was used for analysis based on the p value less than 0.05, 95% C.I. Results*. Among 422 planned respondents, 396 (93.8%) have given the complete response. The mean age of the study population was 27.94 (±5.45). One hundred four (26.26%) out of 396 participants had experienced low back pain in the last 12 months. Driving in sitting upright position OR = 0.32 (95% CI = 0.12–0.86), steer wheel handling OR = 3.02 (95% CI = 1.58–5.77), not holding extra passengers OR = 0.35 (95% CI = 0.21, 0.60), rest breaks, and brand of the three-wheel vehicles were significantly associated with LBP.

**Conclusion:**

Nearly more than one-fourth of three-wheel drivers in our study had LBP. The finding implies a significant number of three-wheel drivers are at risk of developing reduced well-being. An appropriate health visit, lifestyle modification, and adequate policy should be established in the study area.

## 1. Introduction

Work-related low back pain (LBP) is one of the most typical musculoskeletal disorders which affect work performance and the general well-being [1–3]. LBP occurs as a result of muscle tightness beneath the coastal edge and above the lower gluteal pleat [4, 5]. The occurrences of LBP are very common among workers as probably caused, at least in part, or exacerbated by the job climates [4, 5].

**Figure 1:**
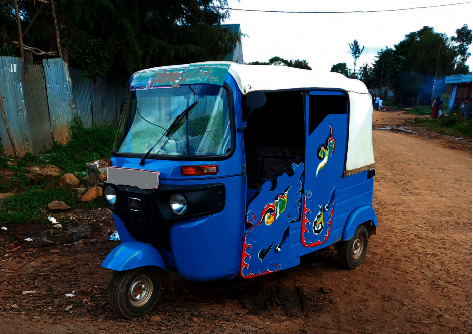
Three-wheel car.

Occupational health has been a major cause of worry in the driving profession particularly among three-wheel drivers [6, 7]. Bajaj is among one of the most important modes of within-city transport system, relatively inexpensive, easily accessible, and an emerging job opportunity in most parts of developing countries [8] (Figure 1).

Globally, 37% of LBP is due to the occupation [9], and its prevalence is rising from 60 to 70% with an incidence rate of 5% [2]. Driving job is often related to the occurrence of LBP due to ergonomic risk factors [10–12]. The prevalence of LBP varies by nation, for example, in USA 81%, Israel 45.4% [12], Sri Lanka 15.5% [13], Malaysia 48.5% [14], Egypt 73.9% [15], and Ethiopia 65% [16].

According to research in the field of ergonomics, prolonged sitting, whole-body vibration, the ergonomic mismatch between anthropometric measurements of the drivers, physical environment, lifting or carrying heavy objects, and prolonged uncomfortable postures while driving are risk factors for LBP [17, 18]. The type of vehicle seat as well as occupational risk factors such as long daily working hours, years of driving, and pressure to compete are the main ergonomic risk factors contributing to LBP [14, 19–21]. However, the extent to which these risk factors are associated with LBP among three-wheel drivers is unknown. Thus, the aim of this study was to identify ergonomic risk factors of LBP among three-wheel drivers in the Jimma city, Ethiopia.

## 2. Methods and Materials

### 2.1. Study Setting and Design

This community-based cross-sectional study was conducted in the Jimma city, Oromia regional state, southwest Ethiopia, from March 01 to March 31, 2020. Jimma city is one of the oldest cities in Ethiopia, and it is located 352 km southwest from the capital Addis Ababa. Based on data from 2016 of the town administration, it has a total population of 195,443. The city has 17 kebeles and two woredas. There are a total of 2000 public three wheel cars in the city that act aspublic transport within 12 site stations, and most of the main roads of the city are covered by asphalt.

### 2.2. Study Population

All four hundred twenty-two selected public three-wheel drivers were our study population. Full-time three-wheel drivers, with at least six months driving experience, on-duty during data collection time, and willing to participate were included in the study. However, individuals who have known LBP prior to entrance into the profession or having a low back pain of traumatic origin were excluded from the study.

### 2.3. Sample Size Estimation

The sample size was estimated using a sample size formula for estimating a single population proportion with a margin of error of 5%, confidence interval of 95%, and by considering a 50% proportion of low back pain among three-wheel drivers because there are no previous data on this study subject. After adding 10% for the nonresponse rate, our final sample size was 422.

### 2.4. Sampling and Data Collection Procedures

The list of three-wheel cars' plate numbers was taken from the city administration with respective terminal sites. Then, three-wheel car plates were randomly selected by computer-generated simple random sampling method using ran between approaches on an excel sheet. Proportional allocation of the total sample size to all terminal's sites (2 sites = 36 samples each and 35 samples on the left 10 of the left terminal sites) was done. A total of 422 three-wheel drivers who fulfil inclusion criteria were interviewed. For observational purposes, a nonparticipatory observational approach was used. Finally, the data were collected using an interviewer-administered questionnaire and observation checklist by trained professionals under consideration of WHO prevention for COVID-19 global pandemic such as mask on, social distancing, and utilization of personal protective methods [22].

Data collection was performed by twelve trained BSc nurses and four supervisors of MSc holders in health discipline by following standard procedures. The pilot test was conducted on 21 participants before the actual data collection period.

### 2.5. Dependent Variable

The dependent variable used in this study was low back pain.

#### 2.5.1. Independent Variables

  Sociodemographic characteristics: age, level of education, ethnicity, religion, marital status, employment status, and average monthly income
  Anthropometric measurements: weight, height, and BMI
  Ergonomics factors: seat, sitting posture, back support, steering wheel, number of passengers beside the driver, rest breaks between driving, work experience, working hour per day, working hour per week, and brand of the three-wheel car
  Low back pain: pain, muscle tension, or stiffness localized below the costal margin and above the inferior gluteal folds, with or without sciatica and is defined as chronic when it persists for 12 weeks or more [23]
  Ergonomic risk factors: workplace situations that cause wear and tear on the body and can cause injury [24]
  BMI: according to our study, it is categorised as underweight (<18.5 kg/m^2^), normal (18.5–24.9 kg/m^2^), overweight (25–29.9 kg/m^2^), and obese (≥30 kg/m^2^) [25]

### 2.6. Data Collection Tool

Data were collected using a standard questionnaire adapted from the Nordic Musculoskeletal Questionnaire (NMQ) and used in different languages in many parts of the world. The NMQ tool's answer confidence ranges from 0 to 23%. Validity tested against clinical history and NMQ found a range of 0 to 20% disagreement [26]. The tool has three domains: domain I sociodemographic characteristics, domain II anthropometric measurements, and domain III Nordic musculoskeletal questions, which contains 7, 4, and 10 items, respectively. Six items of the observational checklist were also used.

After completing the interview, anthropometric measurements (height and weight) were collected. The body weight was measured to the nearest 0.1 kg with a digital weighing scale. The body height was also measured using a steel measuring tape meter to the nearest 0.1 cm from the participant's head to toe in an upright standing position.

### 2.7. Data Analysis Method

Data were analysed using SPSS, version 21.0. Continuous variables were reported as means and standard deviations and categorical variables as counts and percentages.

However, binary logistic regression analysis was used to identify candidate variables, and variables that reached a *p* value <0.25 in the bivariate analysis were entered into the multivariate logistic analysis and the independent predictors of the prevalence of low back pain were estimated considering the *p* value less than 0.05, 95% CI.

## 3. Results

### 3.1. Individual Characteristics of the Participants

A total of 422 drivers were approached, and 396 have given the complete response with 93.84% of response rate. All the study participants were male with different background characteristics. The majority of the participants were young, found in the age range of 21–30 years (64%) with a mean age of 27.94 (±5.45). Nearly three-fourth of the respondents completed the secondary school. About 141 (35.1%) and 255 (64.4%) of respondents were from Orthodox religion and Oromo ethnicity, respectively. Nearly less than one half of the respondents earn between 1500 and 3000 Ethiopian birr per month while a small number 17 (4.3%) earn more than 6000 Ethiopian birr monthly income. Regarding the employment status, more than half of the respondents drive their private three-wheel car and others were employed by the owner of the three-wheel car. More than four-fifth (82.8%) of the respondents' BMIs were ranged between 18.5 and 24.99 kg/m^2^ which is considered as a normal range (Table 1).

### 3.2. Ergonomic Risk Factors and LBP

One hundred four (26.26%) out of 396 participants experienced low back pain within the last 12 months. The three-wheel drivers in the Jimma city reported several ergonomic risk factors such as, uncomfortable seat, sitting posture, back support, and steering wheel with the number of respondents being 118 (29.8%), 136 (34.3%), 148 (37.4%), and 94 (23.7%), respectively, for LBP (Table 2). This result is supported by an observational checklist in which most of the drivers were sitting in an incline position while driving and uncomfortable with steering wheel handling specially when they had held the passenger beside their seat.

However, the finding showed contradictory findings on what aggravates and relieves LBP among those who reported LBP in the last 12 months. While some study subjects reported a sitting position 56 (53.8%), prolonged standing 53 (50.9%), walking 13 (3.3%), side sleep 11 (2.8%), and backward laydown 10 (2.5%) aggravates LBP, on the contrary, the other respondents reported layback ward, side sleep, walking, and sitting with 47 (45.2%), 38 (36.5%), 32 (30.7%), and 12 (11.5%), respectively as relieving factors for LBP (Table 3).

As described in Table 4, comfortable sitting posture, comfortable steering wheel, number of passengers beside the driver, rest breaks between driving, and brand of the three-wheel showed statistically significant association with LBP. Respondents who had comfortable sitting postures were 68% OR = 0.32 [95% CI 0.12–0.86] less likely to develop lower back pain than those who reported uncomfortable sitting posture. Respondents who had no comfortable steer wheel handling were 3 times OR = 3.02 [95% CI = 1.58, 5.77] more likely to experience LBP than respondents who reported their steer wheel handling is comfortable.

Regarding the number of passengers beside the driver, respondents who did not hold passengers beside their seat mostly were 65% OR = 0.35, [95% CI = 0.21, 0.60] more likely to develop low back pain than respondents who were holding passengers beside their seat. Respondents who had a taken a break between driving for less than 15 minutes and 15 minutes and more were less likely to develop LBP than respondents who had not a break between driving OR = 0.45 [95% CI = 0.21, 0.94] and OR = 0.31 [95% CI = 0.12, 0.79], respectively. Respondents who were driving brand 2 and brand 4 of the three-wheel car were more likely to develop lower back pain than respondents who were driving brand 5 OR = 10.19 [95% CI = 0.91, 113.62] and OR = 9.37 [95% CI = 1.13, 77.63], respectively (Table 4).

## 4. Discussion

This study investigated the association between work-related ergonomic risk factors and LBP among three-wheel drivers in the Jimma city, southwest Ethiopia. The finding showed that 26.26% of the three-wheel drivers experienced LBP in the last 12 months. This may indicate driving three-wheel car subjects the individual to the tightness of the muscle beneath the coastal edge and lower gluteal pleat [4, 5]. Other studies have also reported the episodes of LBP are high in such working environments [10–12].

The current study identified that uncomfortable sitting postures, steering wheel handling, number of passengers beside the driver, rest breaks between driving, and brand of the three-wheel car as significant risk factors for experiencing LBP among three-wheel drivers. These findings are in line with reports from prior studies [14, 19–21]. This indicates that the work environment exposed them to the ergonomic risk factors which in turn risked them to LBP.

This finding shows that drivers who reported comfortable seat posture were 68 times less likely to develop low back pain than respondents who had an uncomfortable sitting posture. This finding is similar to studies conducted in Malaysia [19] and Ethiopia [16]. The current findings also revealed that respondents who had taken a break between driving were less likely to develop LBP than respondents who had not taken a break between driving. This finding is similar to studies from Israel [5] and Ethiopia [20].

Furthermore, respondents who complained of uncomfortable steer wheel handling were 3 times more likely to develop LBP than respondents who did not report uncomfortable steer wheel handling. This is comparable with a study conducted in Egypt [15] in which drivers who complained of uncomfortable steering wheels revealed a high prevalence of lower back pain.

### 4.1. Strengths and Limitations

The main strengths of this study are that we used a nonparticipatory observation checklist of records as confirmatory for self-reported ergonomic risk factors for LBP. Moreover, this study gives attention to the occupation-related health problem of three-wheel drivers which was neglected by the health care workers and researchers. In addition, the data collection was performed under consideration of WHO prevention for COVID-19 global pandemic.

However, the limitations of this study include, like any observational study, the analysis might only identify the associations between exposure to risk factors and LBP but not fix the causality. The twelve months work-related lower back pain prevalence may be under or overestimated due to recall bias. Therefore, the recall bias might be another weakness of the study. Thus, for future, we recommend a longitudinal study for better outcome in all dimensions of pain assessment. This study was conducted in only one part of southwest of Ethiopia which may not show a more comprehensive result representing three-wheel drivers working in Ethiopia.

## 5. Conclusion

This finding shows that nearly more than one-fourth of three-wheel drivers of the Jimma city had low back pain. Some ergonomic risk factors such as uncomfortable sitting postures, steer wheel handling, number of passengers beside the driver, rest breaks between driving, and brand of the three-wheel car are significantly associated with LBP among three-wheel drivers. The finding implies a significant number of three-wheel drivers are at risk of developing reduced well-being. An appropriate health visit, lifestyle modification, and adequate policy brief that addresses this high burden occupation-related problem should be established in the study area.

## Figures and Tables

**Table 1:** Sociodemographic characteristics among three-wheel drivers in the Jimma city, southwest Ethiopia, 2020.

Variable	Frequency	Percentage
*Age (in years)*		
Less than 20	30	7.6
20–30	254	64.1
31–40	102	25.8
≥41	10	2.5
*Educational level*		
Primary school	60	15.2
Secondary school	261	65.9
College and above	75	18.9
*Marital status*		
Single	227	57.3
Married	163	41.2
Widowed	6	1.5
*Religion*		
Muslim	130	32.8
Orthodox	141	35.6
Protestant	125	31.6
*Ethnicity*		
Oromo	255	64.4
Amhara	46	11.6
Kefa	39	9.8
Yem	17	4.3
Dewaro	18	4.5
Gurage	10	2.5
Wolayita	11	2.8
*Average monthly income (Ethiopian birr)*		
<1500	51	12.9
1500–3000	182	46.0
3001–6000	146	36.9
>6000	17	4.3
*Body mass index (kg/m * ^*2*^ * )*		
<18.5	28	7.1
18.5–24.99	328	82.8
25–29.99	36	9.1
≥30	4	1.0
*Car ownership*		
Owner	207	52.3
Employee	189	47.7

**Table 2:** Ergonomic risk factors related to the car among three-wheel drivers.

Variable	Frequency	Percentage
*Comfort seat*		
No	118	29.8
Yes	278	70.2
*Comfortable sitting postures*		
No	136	34.3
Yes	260	65.7
*Comfortable back support*		
No	148	37.4
Yes	248	62.6
*Comfortable steering wheel*		
No	94	23.7
Yes	302	76.3
*Hold extra passengers*		
No	290	73.2
Yes	106	26.8
*Brand of the three-wheel car*		
Brand 1	230	58.1
Brand 2	13	3.3
Brand 3	6	1.5
Brand 4	133	33.6
Brand 5	48	12.1

**Table 3:** Work-related risk factors among three-wheel drivers.

Variable	Frequency	Percentage
*Work experience*		
Less than 5 years	356	89.9
Five year and more	40	10.1
*Daily working hours*		
8 hours and less than 8	141	35.6
More than eight hours	255	64.4
*Working days per week*		
5 and less than 5 days	14	3.5
More than five days	382	96.5
*Rest breaks between driving*		
None	48	12.1
Less than 15 minutes	67	16.9
≥15 minutes	281	71.0
*Age (in years)*		
Less than 20	30	7.6
20–30	254	64.1
31–40	102	25.8
≥41	10	2.5
*Educational level*		
Primary school	60	15.2
Secondary school	261	65.9
College and above	75	18.9

**Table 4:** Bivariate and multivariate logistic regression model showing ergonomic predictors of LBP among three-wheel drivers.

Variables	COR/95% CI/*P* value	AOR/95% CI/*P* value
*Comfort seat*		
No	0.61 (0.36, 1.04)/0.07^*∗*^	1.29 (0.48, 3.46) 0.60
Yes	1	1
*Comfortable sitting postures*		
Yes	0.52 (0.32, 0.90) 0.01^*∗*^	0.32 (0.12, 0.86) 0.02^*∗∗*^
No	1	1
*Comfortable back support*		
No	0.86 (0.54, 1.38) 0.55	
Yes	1	
*Comfortable steering wheel*		
No	1.47 (0.88, 2.44) 0.13^*∗*^	3.02 (1.58, 5.77) 0.00^*∗∗*^
Yes	1	1
*Do you hold passengers beside you mostly?*		
No	0.43 (0.26, 0.69) 0.00^*∗*^	0.35 (0.21, 0.60) 0.00^*∗∗*^
Yes	1	1
*Work duration by year*		
Less than 5 years	0.43 (0.22, 0.84) 0.01^*∗*^	0.56 (0.27, 1.19) 0.13
Five year and more	1	1
*Daily working hours*		
8 hours and less than 8	0.76 (0.47, 1.22) 0.26	
More than eight hours	1	
*Working days per week*		
5 and less than 5 days	0.46 (0.10, 2.10) 0.32	
More than five days	1	
*Rest breaks between driving*		
None	1	1
Less than 15 minutes	0.44 (0.22, 0.89) 0.02^*∗*^	0.45 (0.21, 0.94) 0.03^*∗∗*^
15 minutes and more	0.32 (0.13, 0.79) 0.01^*∗*^	0.31 (0.12, 0.79) 0.01^*∗∗*^
*Brand of the three-wheel car*		
Brand 1	4.38 (0.56, 34.2)/0.16^*∗*^	6.45 (0.78, 53.05) 0.08
Brand 2	8.12 (0.79, 82.7) 0.07^*∗*^	10.19 (0.91, 113.62) 0.05^*∗∗*^
Brand 3	0.00 (0.00)/0.99	0.00 (0.00) 0.99
Brand 4	5.99 (0.68, 42.65)/0.11^*∗*^	9.37 (1.13, 77.63)/0.03^*∗∗*^
Brand 5	1	1

^*∗*^Significant at *P* value ≤0.25; ^*∗∗*^significant at *P* value ≤0.05.

## Data Availability

The data that support the findings of this study are available upon request from the corresponding author, Yonas Biratu. The data are not publicly available due to their containing information that could compromise the privacy of research participants.
